# Evaluating cell viability, capillary perfusion, and collateral tortuosity in an ex vivo mouse intestine fluidics model

**DOI:** 10.3389/fbioe.2022.1008481

**Published:** 2022-12-09

**Authors:** Caroline E. Willi, Hanaa Abdelazim, John C. Chappell

**Affiliations:** ^1^ Fralin Biomedical Research Institute (FBRI) at Virginia Tech-Carilion (VTC), Roanoke, VA, United States; ^2^ FBRI Center for Vascular and Heart Research, Roanoke, VA, United States; ^3^ Department of Biomedical Engineering and Mechanics, Virginia Tech, Blacksburg, VA, United States; ^4^ Department of Basic Science Education, Virginia Tech Carilion School of Medicine, Roanoke, VA, United States

**Keywords:** pericytes, vascular stability, reperfusion injury, collateral flow, microfluidic models

## Abstract

Numerous disease conditions involve the sudden or progressive loss of blood flow. Perfusion restoration is vital for returning affected organs to full health. While a range of clinical interventions can successfully restore flow to downstream tissues, the microvascular responses after a loss-of-flow event can vary over time and may involve substantial microvessel instability. Increased insight into perfusion-mediated capillary stability and access-to-flow is therefore essential for advancing therapeutic reperfusion strategies and improving patient outcomes. To that end, we developed a tissue-based microvascular fluidics model to better understand (i) microvascular stability and access-to-flow over an acute time course post-ischemia, and (ii) collateral flow in vessels neighboring an occlusion site. We utilized murine intestinal tissue regions by catheterizing a feeder artery and introducing perfusate at physiologically comparable flow-rates. The cannulated vessel as well as a portion of the downstream vessels and associated intestinal tissue were cultured while constant perfusion conditions were maintained. An occlusion was introduced in a selected arterial segment, and changes in perfusion within areas receiving varying degrees of collateral flow were observed over time. To observe the microvascular response to perfusion changes, we incorporated (i) tissues harboring cell-reporter constructs, specifically *Ng2-DsRed* labeling of intestinal pericytes, and (ii) different types of fluorescent perfusates to quantify capillary access-to-flow at discrete time points. In our model, we found that perfusion tracers could enter capillaries within regions downstream of an occlusion upon the initial introduction of perfusion, but at 24 h tissue perfusion was severely decreased. However, live/dead cell discrimination revealed that the tissue overall did not experience significant cell death, including that of microvascular pericytes, even after 48 h. Our findings suggest that altered flow conditions may rapidly initiate cellular responses that reduce capillary access-to-flow, even in the absence of cellular deterioration or hypoxia. Overall, this *ex vivo* tissue-based microfluidics model may serve as a platform upon which a variety of follow-on studies may be conducted. It will thus enhance our understanding of microvessel stability and access-to-flow during an occlusive event and the role of collateral flow during normal and disrupted perfusion.

## Introduction

Blood vessels facilitate nutrient exchange and waste removal to sustain the health of all tissues. When these essential conduits become obstructed such as in stroke, myocardial infarction, or acute intestinal ischemia (e.g., in necrotizing enterocolitis), it is critical to re-establish perfusion and restore organ homeostasis (Nowicki, 2005; Moskowitz et al., 2010). Clinical treatments for stroke, for instance, include rapid infusion of tissue plasminogen activator (tPA, chemical thrombus disaggregation) within 4 h last-known-well, or catheter-based mechanical thrombectomy (MT, surgical clot removal) in the hyper-acute phase between 4 h and recently-extended 24 h last-known-well (Powers et al., 2018). Similar approaches are employed in other acute ischemia scenarios such as myocardial infarction, but tissue reperfusion outcomes can vary widely and suffer from the “no-reflow” phenomenon, that is, the incomplete restoration of blood flow to all capillary beds (Eeckhout and Kern, 2001; Ito, 2006). A more complete understanding of the mechanisms that limit reperfusion will therefore inspire the development of next-generation therapies that can achieve better recovery endpoints.

Blood vessels are known to experience reperfusion injury when flow is reintroduced to vasculature following static conditions. This complex scenario involves the convergence of mechanical and signaling mechanisms that yield a range of consequences including increased leukocyte recruitment, disrupted vasomotion, and microvessel obstruction and instability (Heusch, 2020). Circulating thrombi may lodge within microvascular networks and reduce blood flow to capillary beds (Bray et al., 2020), and reactive oxygen species induce cellular damage, dysfunction, and death (Granger and Kvietys, 2015). It has also been proposed that vascular pericytes in the heart and brain may constrict capillaries during ischemia and die in this constricted state, resulting in permanently occluded microvessels (Yemisci et al., 2009; Hall et al., 2014; O'Farrell and Attwell, 2014; O'Farrell et al., 2017). Recent observations have questioned the extent to which dead pericytes may remain intact and are able to sustain capillary occlusion (Hoque et al., 2021), but pericyte loss from the vessel wall is likely a key component of microvessel instability that may limit tissue reperfusion. While our knowledge of the underlying causes for inadequate perfusion after recanalization continues to expand, many open questions remain regarding post-ischemia capillary access-to-flow and stability. These gaps in knowledge highlight the need for additional experimental models that might help dissect molecular and biophysical factors contributing to restricted blood flow distribution following therapeutic intervention.

A range of experimental models have been developed to gain insight into specific aspects of tissue ischemia and reperfusion injury responses. *In vivo* models frequently involve vessel occlusion by ligation or clamp for a short period of time and then removing the occlusion to reestablish blood flow to the downstream tissue (Gonzalez et al., 2015). These models capture inherent biological complexities within the ischemia-reperfusion setting including the cellular, biochemical, and mechanical components of blood as well as the effects of hypoxia, among other factors (Heusch, 2020). Conversely, these complexities can pose challenges with interpreting results, especially when overlaid on other variables that are altered due to the experimental approach e.g., surgical intervention causing systemic immune activation and anesthesia altering cardiovascular physiology (Luo et al., 2007). *In vivo* models are also restricted temporally, not easily facilitating experiments that correspond to the timing of ischemia onset and clinical intervention.

In contrast, cell-based microfluidic models allow for flexibility in cell type selection to mirror specific vasculature of interest such as the blood-brain barrier (BBB) (Bang et al., 2017), placental exchange (Haase et al., 2019), or the tumor microenvironment (Phan et al., 2017). These models often incorporate primary cells e.g., human umbilical vein endothelial cells (HUVECs) or differentiated stem cells (embryonic or induced pluripotent), and can achieve expression of vascular markers and comparable physiological behaviors (Song and Munn, 2011; Akbari et al., 2017). Nevertheless, they often lack important blood-derived inputs such as key fluid mechanics and circulating hormonal signals as well as the complexity of a tissue-specific microenvironment i.e., adjacent cell types and extracellular matrix (ECM) components. These experimental platforms are also often limited by (i) a paucity of vessels that remain capillary size (i.e., diameters less than 10 μm) and (ii) heterogeneous mural cell incorporation such as by pericytes and/or smooth muscle cells (Hesh et al., 2019). To bridge *in vivo* and *in vitro* models, *ex vivo* models have been developed as an intermediate approach for retaining many important biological features while allowing greater experimental control and more rigorous assessment, though with inherent limitations as well.

Tissue-based *ex vivo* models typically involve the extraction of an organ of interest and preparing it to be cultured in conditions that sustain cellular health and minimize deviation from the *in vivo* scenario. New insights into the dynamics of vascular remodeling and barrier function have been gleaned from *ex vivo* models of retinal (Sawamiphak et al., 2010), mesenteric (Azimi et al., 2017; Suarez-Martinez et al., 2018), lung (Sava et al., 2017), and dermal (Payne et al., 2021) microcirculatory networks, though fluid transit was absent in these models. To capture the effects of intraluminal forces on the vessel wall, a subset of tissue-based models have incorporated pressurization of the vasculature as an orthogonal approach to better understand vasomotor responses to tissue activities and behavior (Hall et al., 2014). Recently, a number of perfused *ex vivo* models have been developed to recapitulate aspects of fluid mechanics on blood vessel physiology (Goeden and Bonnin, 2013; Motherwell et al., 2019; Gonzales et al., 2020). Although these models lack key features such as the viscosity and cellular or biochemical components of blood, they retain critical aspects of vessel wall structure and composition (e.g., ECM components) in addition to the intrinsic architecture and hierarchy of *in vivo* vascular networks.

In the current study, we sought to develop an *ex vivo* fluidics platform utilizing the mouse intestinal vasculature, inspired by recently developed approches involving rat mesentery (Motherwell et al., 2019). We sought to address the question of why capillary perfusion may become restricted in vessel occlusion and ischemia-reperfusion scenarios. Our specific focus was on the influence of cell viability and fluid mechanics on flow through capillary networks. As such, we established culture conditions that sustained tissue viability and allowed a closed-loop perfusion of intestinal vessels for up to 48 h. To model ischemic injury, a vessel blockage was simulated by an occlusion of an arterial branch downstream of the catheterization point. We verified that perfusate could access intestinal capillaries within 1 h of experimental set up, including those downstream of the occlusion. Nevertheless, after 24 h and 48 h of sustained fluid flow *ex vivo*, the microvasculature lacked perfusion when assessed by FITC-Dextran-mediated fluorescence angiography. We also found that secondary intestinal arteries displayed a higher degree of tortuosity at both time points, suggesting these vessels experienced higher intraluminal pressures that could not be resolved by fluid flow through downstream capillary networks. Overall, our results suggest that restricted capillary perfusion may be less affected by microvessel stability than previously thought, as cell viability, specifically that of the pericytes, was largely maintained, and yet microvessel fluid flow rapidly decreased. Our study also reinforces the potential utility of *ex vivo* fluidics platforms in addressing open questions regarding the microvascular response to changes in tissue perfusion.

## Materials and methods

### Animal use

All animal experiments were conducted with review and approval from Virginia Tech Institutional Animal Care and Use Committee (IACUC), which reviewed and approved all protocols. The Virginia Tech NIH/PHS Animal Welfare Assurance Number is A-32081–01 (expires 31 July 2025). To view pericytes on intestinal capillaries, mice expressing the red fluorescent protein variant (DsRed.T1) under control of the mouse NG2 (*Ng2/Cspg4*) promoter/enhancer [i.e., *Ng2:DsRed* mice–NG2DsRedBAC, Stock name Tg (Cspg4-DsRed.T1) 1Akik/J, #008241, The Jackson Laboratory] were utilized between the ages of 6 and 12 months old. Wild type mice were also used, specifically C57BL/6 mice (Stock name C57BL/6J, #000664, Jackson Laboratory) within the same age range (see Supplementary Material for more details).

### Perfused ex vivo intestial tissue preparation

Murine intestinal tissue was excised and transferred to a tissue culture dish. The incision points were sutured and cleared of debris. The area of interest was established by identifying a section with (i) a relatively large artery to ease needle insertion, and (ii) downstream branching that allows for the introduction of an occlusion to a central branch while neighboring branches could remain non-occluded and sustain flow. Sutures were made at the occlusion site, around neighboring, non-catheterized arteries, and on the intestines at either end of the area of interest. The catheter was introduced by making an incision in the feeder artery and gently advancing a 33G needle connected to silicone tubing into the vessel. The connection was secured with tissue adhesive, and the tissue was transferred to an incubator. The catheter was connected to a peristaltic pump, which administered pulsatile flow (1 ml/min–1.5 ml/min flow rate). The peristaltic pump functions by alternating between compressing and decompressing flexible tubing containing fluid as the rotor rotates. This motion creates pockets of fluid that quickly form as the rotor position moves from compressing to releasing. This quick fluid motion/tubing expansion creates a pulse. To establish a closed-loop system, the same solution acting as the perfusate was also used as tissue culture media. Perfusate solution/media is Minimum Essential Medium (Thermo Fisher catalog #11095080) with 1% penicillin streptomycin and fetal bovine serum (see Supplemental Material). The incubation media volume was controlled by a secondary pump that removed excess volume and deposited it back into the reservoir for reperfusion. Controlled flow was maintained throughout the duration of tissue culture.

### Sample immunostaining, imaging, and analysis

Experiments were completed over 1 h, 24 h, and 48 h to assess the tissue and vascular responses at these time points. Immediately following the cessation of pump-administered controlled flow, the tissue was perfused with a FITC-Dextran solution and imaged using a Zeiss Discovery dissection microscope. The tissue was then moved to a Zeiss Epi-Fluorescent microscope to image vessel tortuosity. Finally, the tissue was stained unfixed using the live-dead stain DRAQ7 with subsequent labeling by DAPI or Hoechst to detect cell nuclei and imaged on a Zeiss LSM 880 confocal microscope.

All image quantification was conducted using ImageJ/FIJI software (Rasband, 1997; Schindelin et al., 2012), and, aside from the pericyte viability assay, biological replicates for each experiment were at a minimum of *n* = 3. Cell viability was quantified as the percentage of DRAQ7+ and DAPI+ or Hoechst+ cells relative to the total number of DAPI+ or Hoechst+ cell nuclei. Positivity for DRAQ7 is indicative of permeable cells that are non-viable due to apoptosis or necrosis. Integrated density measurements of FITC-Dextran (measurement area x mean of gray values) was captured at three locations in each of the five distinct areas relative to the occluded area, as well as a background value for each image. The background value was subtracted from each metric to account for differences in imaging parameters, yielding the relative integrated density value. The distance-metric tortuosity was quantified by (i) measuring the displacement distance from vessel end-points, and (ii) dividing that value by the direct-line distance. Tortuosity was measured for vessels classified by diameter as “primary” (greater than 45 μm in diameter) and secondary (less than 45 μm in diameter).

### Statistical analysis

Statistical analysis was performed in GraphPad Prism 8 software. For all measurements, we applied an ordinary one-way Analysis of Variance (ANOVA) test followed by Tukey’s multiple comparisons test to analyze differences between each experiment group and/or classification. Statistical significance was achieved when *p* ≤ 0.05. Again, all experiments were conducted with an *n* = 3 or more for biological replicates (with a biological replicate being one individual animal), and technical replicate measurements were taken per each biological replicate.

## Results

### Mouse intestinal vasculature can be excised and cultured while being perfused

**FIGURE 1:**
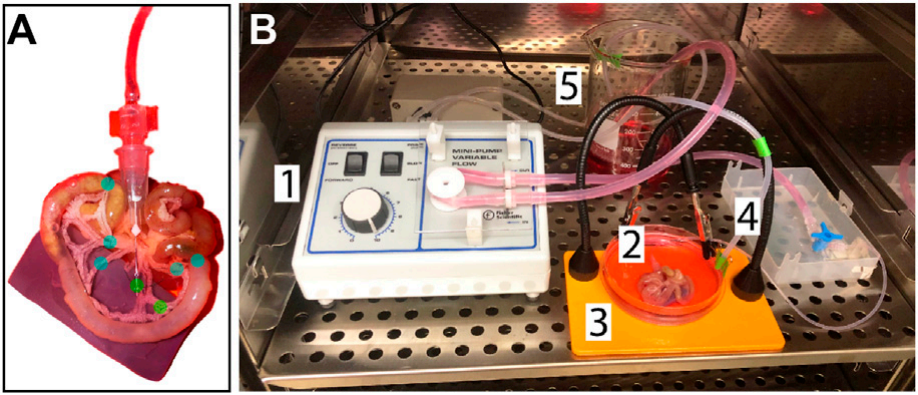
Experimental Design and Setup Schematics. **(A)** Close-up of the catheter insertion point and suture points: The green dots label the needle insertion point, which is secured with tissue adhesive, and the occlusion suture. The blue dots label sutures made on neighboring, non-catheterized arteries and on the intestine on either end of the area of interest. These sutures serve to emulate the resistance that would be present *in-vivo* from blood flow. **(B)** Set up of the controlled-flow tissue culture environment: 1. The peristaltic pump provides controlled flow of the perfusate (MEM with 1% penicillin-streptomycin and 10% fetal bovine serum) at a flow-rate of 1 ml/min *via* an arterial catheter, 2. 3. The tissue is kept in the same media solution acting as the perfusate to create a closed circuit where the media volume in the dish is controlled by a secondary pump, 4, and deposited back into the reservoir to be cycled again, 5. All components are maintained at 37°C and 5% CO_2_.

In an effort to develop an intermediate platform bridging *in vitro* microfluidic approaches and *in vivo* experimental models, we drew from previous studies suggesting the mouse intestinal vasculature as an organ system that could be excised and cultured *ex vivo* similar to rat-based models (Azimi et al., 2017; Suarez-Martinez et al., 2018; Motherwell et al., 2019). We found vessel segments downstream of the superior mesenteric artery to be accessible *via* a 33G needle after minimal resection of adipose and connective tissue (Figure 1). Initial attempts at perfusion revealed the necessity for occlusion of non-catheterized arteries in flanking regions to simulate intraluminal fluid pressure and prevent retrograde flow back up these vessels. This configuration resulted in (i) fluid flow largely, though not completely, out of veins, and (ii) post-transit perfusate contributing to the media surrounding the isolated tissue. To achieve a closed-loop system, culture media was pumped into the perfusion reservoir that continuously flowed through the intestinal vasculature *via* peristaltic pump (Figure 1). The entire platform was placed in an incubator to maintain temperature (37°C), humidity, and an appropriate gas environment (5% CO_2_) for the media selected.

**FIGURE 2:**
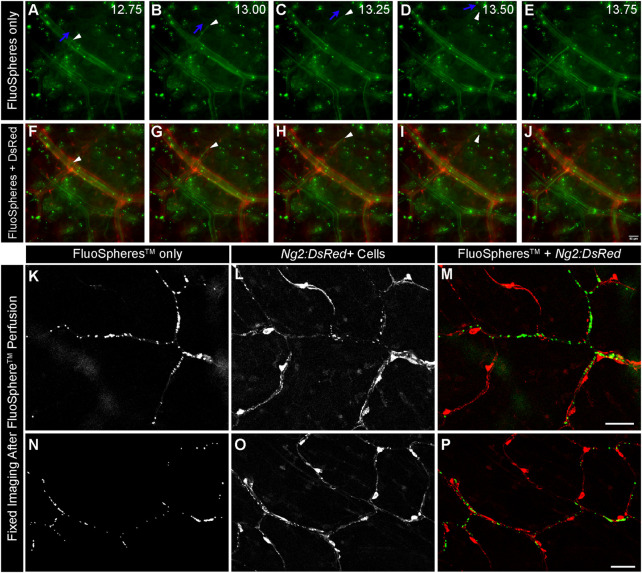
Green FluoSpheres^TM^ Perfuse *Ng2:DsRed*-labeled Intestinal Capillaries. Representative time-lapse images of fluorescent green microspheres [green in **(A–J)**, 1 μm in diameter] suspended in PBS traveling through the intestinal vasculature *via* feeder artery catheterization in an *Ng2:DsRed* reporter mouse [red, in **(F–J)**]. An artery-vein pair is shown, in which the vessels are distinguished by the presence of DsRed in the NG2+ smooth muscle cells wrapped around the artery **(F–J)**. Green microspheres are observed traveling through both the artery and the vein, validating that the spheres are advancing down to the microvasculature. The directionality of one sphere is labeled with blue arrows, and the sphere is indicated by a white arrowhead in panels **(A–D)**. Capturing the phenomenon of a single 1 μm sphere flowing through a smaller-order vessel further supports the idea that our mechanism of providing flow is able to advance to the microvascular level. Time is shown in seconds **(A–E)**. Scale bar in **(J)** is 50 μm. Representative confocal images of fluorescent microspheres [**(K,N)** green in **(M,P)**] adjacent to *Ng2:DsRed+* pericytes [**(L,O)** red in **(M,P)]** after perfusion through the intestinal capillaries. Scale bars in **(M,P)** are 50 μm.

One primary goal in developing this system was to model fluid flow through capillary networks (i.e., vessel diameter less than 10 um, pericytes present, established ECM basement membrane, etc.), as this remains elusive in the design of microfluidic platforms (Hesh et al., 2019). We perfused PBS systemically *via* an intracardiac catheter following euthanasia to limit thrombi formation that might prevent flow through the intestinal microcirculation. We avoided reintroducing flow abruptly following artery cannulation to reduce the likelihood and/or severity of reactive vasoconstriction that might also restrict perfusion within the microvasculature. To confirm that these approaches were useful in maintaining capillary access to fluid flow, we introduced green fluorescent microspheres with 1 μm diameters (FluoSpheres™) into the perfusate. We then used live imaging to capture their transit through microvessels surrounded by *Ng2:DsRed+* pericytes (Figure 2). Microspheres rapidly flowing through intestinal vessels appeared as streaks of light indicating their path of travel through vessel networks. We noted numerous instances of microspheres passing through capillary-sized vessels clearly associated with *Ng2:DsRed+* pericytes (Figure 2). Further evidence of capillary transit was observed in high-power confocal images acquired after microsphere perfusion in which FluoSpheres™ were found intraluminally adjacent to *Ng2:DsRed+* pericytes (Figure 2) and not outside of the vessel wall. Taken together, this data instills confidence that capillary perfusion was maintained during experimental set up.

### Static and controlled flow ex vivo culture conditions maintain tissue viability over 24 h and 48 h time courses

**FIGURE 3:**
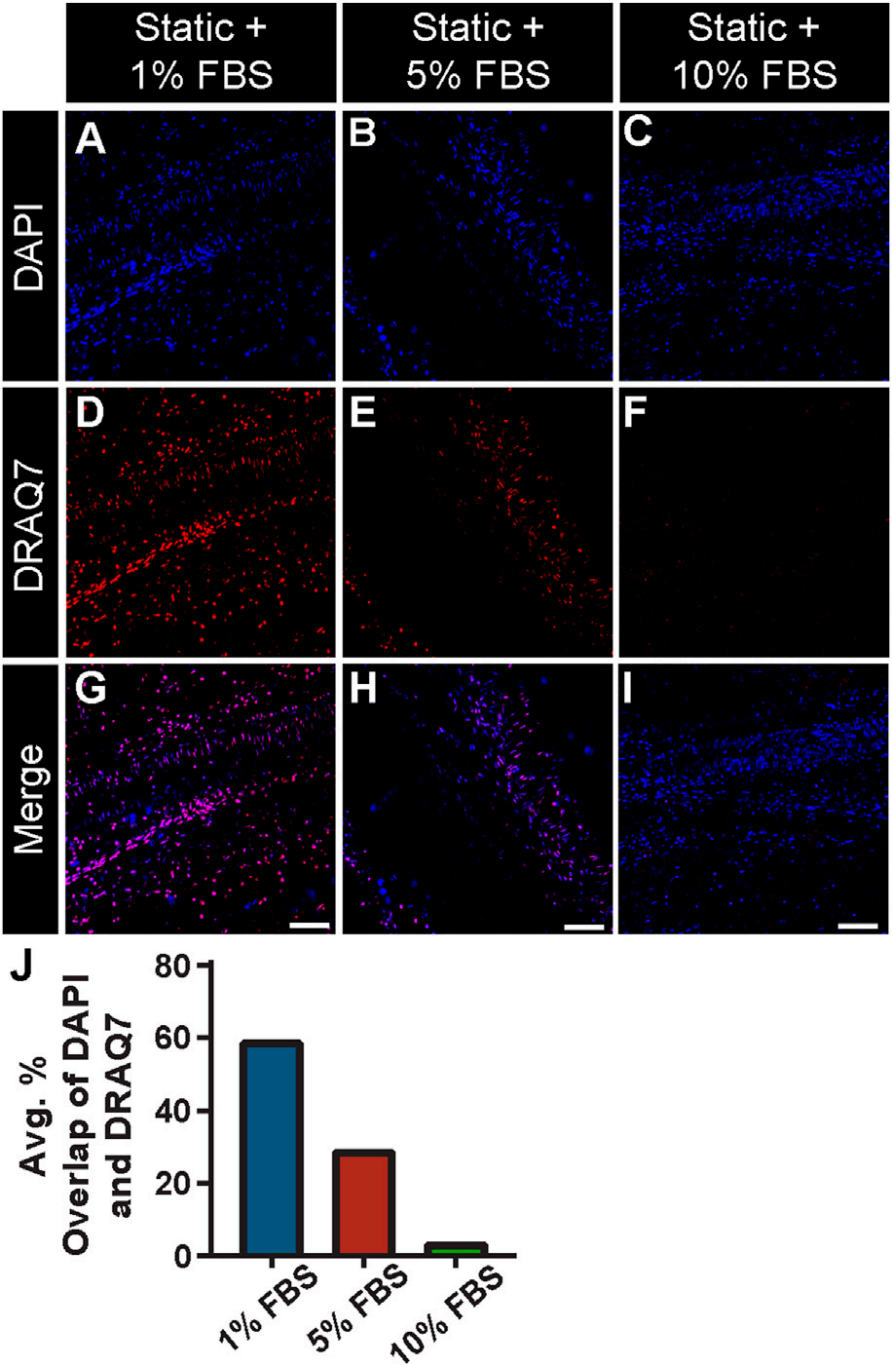
FBS Concentration Assessment with a Cell Viability Assay under Static Conditions. Representative images of DAPI-labeled cell nuclei [blue in **(A–C)** and **(G–I)**] relative to compromised cell nuclei labeled with DRAQ7 [red in **(D–I)]** in a 48 h static tissue culture of intestinal sections with 1% **(A,D,G)** 5% **(B,E,H)** and 10% FBS **(C,F,I)** in media. Note, 10% FBS showed a significant reduction of cell death compared to 5% and 1%, with the percent overlap of DAPI and DRAQ7 found to be 54.58%, 15.60%, and 1.82% for 1%, 5%, and 10% FBS concentrations, respectively. Therefore, 10% FBS was used in the experimental setup with controlled flow. Scale Bars in **(G,H,I)** are 100 μm. **(J)** Average percent overlap of DAPI and DRAQ7 signals across the intestinal tissue area under static conditions with 1% FBS (blue bar), 5% FBS (red bar), and 10% FBS (green bar).

Previous studies have suggested that the viability of cells within the microcirculation plays a critical role in vessel stability and in turn perfusion of microvascular networks (Galiuto et al., 2000). Specifically, pericytes have been described as severely narrowing capillary diameters and dying in this constricted state, thereby limiting downstream blood flow and exacerbating the no-reflow phenomenon (Yemisci et al., 2009; Hall et al., 2014; O'Farrell et al., 2017). Therefore, a critical aspect in developing this experimental setup was establishing the appropriate culture conditions that maintained tissue viability over an extended period without activating cells (i.e., inducing proliferation, etc.). We tested a range of concentrations for fetal bovine serum (FBS) under static conditions and found that 1% and 5% were insufficient in limiting cell death. Specifically, we detected a relatively high number of cells labeled by DRAQ7, which was used to identify permeabilized, non-viable cells due to the ability of this dye to only access the nuclei of dying cells (Figure 3). We suspect cell death was largely due to necrosis, though apoptotsis may also be occurring. In contrast, 10% FBS significantly lowered the presence of DRAQ7+ cells, suggesting that while some cell death was occurring in these intestine preparations, a higher concentration of serum was capable of minimizing cell loss and tissue degradation.

**FIGURE 4:**
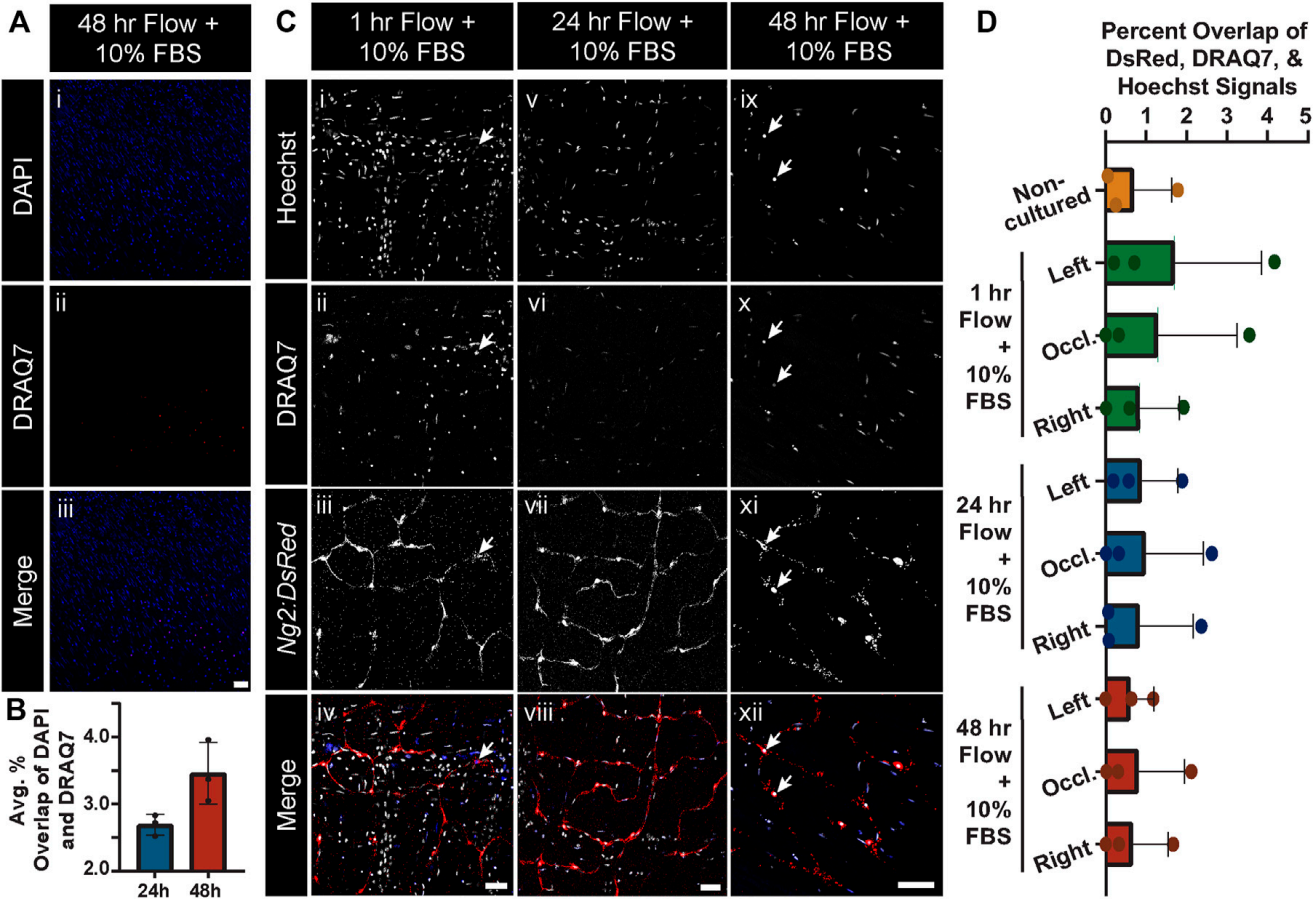
Global and *Ng2:DsRed+* Cell Viability Remains Unchanged after Culture in Controlled Flow Conditions. **(A)** Representative images of general cell viaibility as gauged by DAPI-labeled cell nuclei (blue in i and iii) relative to compromised cell nuclei labeled with DRAQ7 (red in ii and iii) in a 48 h tissue culture with 10% FBS and controlled flow. Scale bar in iii is 50 μm. **(B)** Average percent overlap of DAPI and DRAQ7 signals across the intestinal tissue area at 24 h (blue bar) and 48 h (red bar) where controlled flow was supplied (note, *y*-axis range is from 2% to 4.5%). Error bars represent ± standard deviation. Note, the live-dead staining with DRAQ7 revealed that there was not extensive cell death under the controlled flow and tissue culture conditions at 24 h or 48 h, despite the lack of flow access to most smaller-order vessels. Biological replicates for this experiment were at a minimum of *n* = 3. **(C)** Representative images of Hoechst-labeled cell nuclei (i, v, ix; white in iv, viii, xii), DRAQ7-labeled cell nuclei (ii, vi, x; blue in iv, viii, xii), and *Ng2:DsRed*-labeled pericytes (iii, vii, xi; red in iv, viii, xii) in the occluded areas of 1 h (i-iv), 24 h (v-viii), and 48 h (ix-xii) cultures with controlled flow. Scale bars in (iv), (viii), (xii) are 50 μm. Note, live-dead staining with DRAQ7 revealed no notable pericyte death, even in the occluded area. Additionally, no abnormal pericyte morphology was observed. **(D)** Average percent overlap of DsRed, DRAQ7, and Hoechst signals across intestinal tissues not cultured (orange bar and circles) and cultured in controlled flow conditions for 1 h (green bars and circles), 24 h (blue bars and circles), and 48 h (red bars and circles). Error bars represent standard deviation.

While 10% FBS in the culture media appeared sufficient in limiting cell death and tissue degradation during static conditions, we sought to address the question of maintaining cell viability under flow conditions and over an extended culture time course. After 24 h and 48 h of culture with flow maintained at physiologically relevant levels, we assessed intestinal tissue viability by incubating with DRAQ7, again a dye that should not label cell DNA extensively unless the nuclear envelope is compromised, potentially indicative of progression towards cell death. At 24 h, we found on average approximately 2.6% of all cells positive for DRAQ7, with only a slight increase to 3.5% at the 48 h time point (Figure 4). We also noted that vascular cell types such as pericytes were sustained by this approach because we observed very little, if any, overlap in the *Ng2:DsRed+* signals and DRAQ7 labeling (Figure 4) nor did we find gross changes in microvessel morphology (see Supplementary Figure S1). Some disruption in the continuity of the DsRed signal was noted, which may be attributable to early stages of cell degeneration but also to stability dynamics for this particular fluorescent reporter (Sacchetti et al., 2002). These data suggest that flow conditions did not severely diminish cell viability at the time points observed, suggesting further that the media circulating within and around isolated tissues was able to sustain a relatively high level of cell and tissue viability over the experimental time course.

### Perfusion of intestinal capillary networks was limited after 24 h and 48 h despite lack of tissue degradation

**FIGURE 5:**
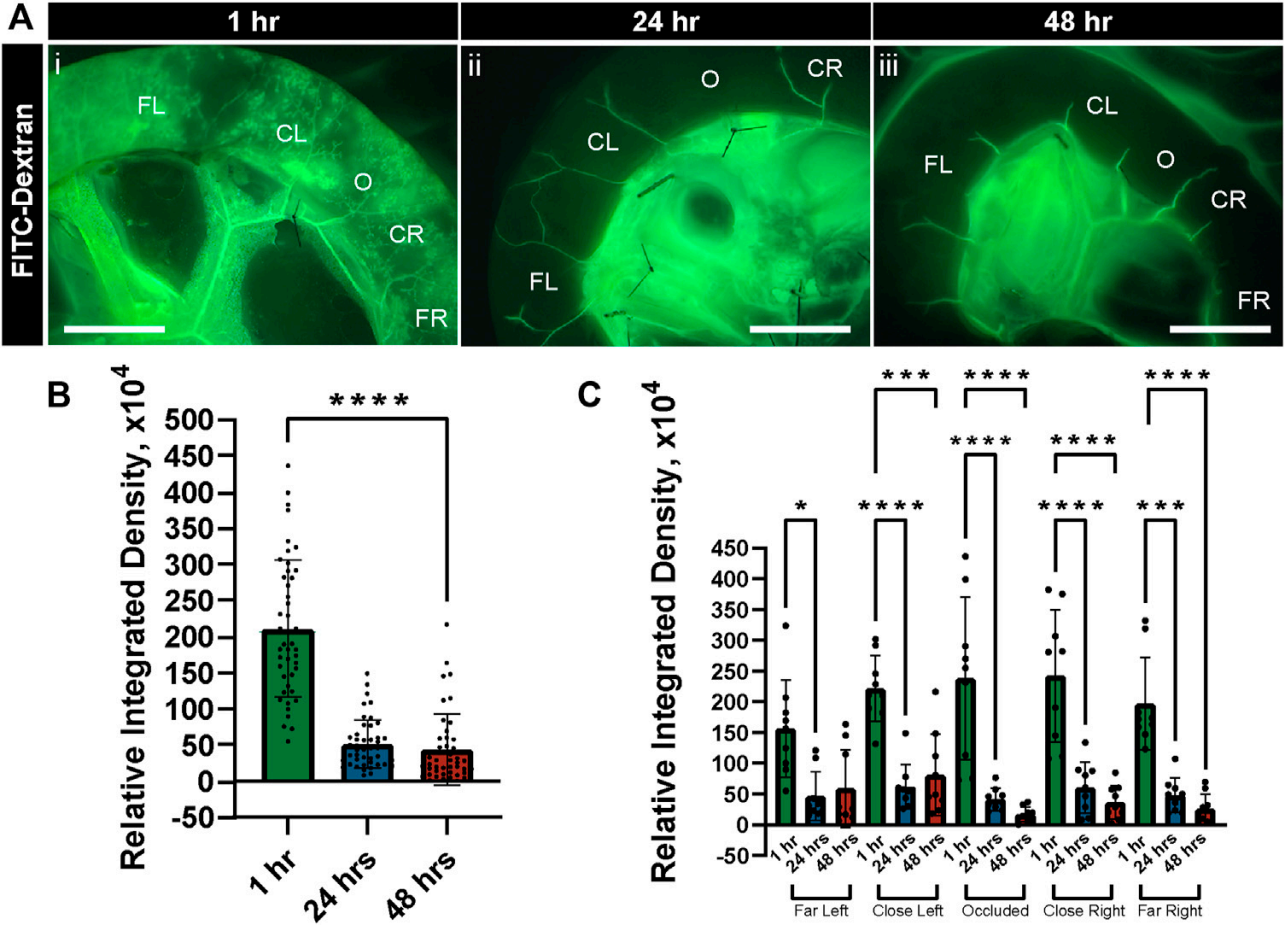
FITC-Dextran Perfusion in Intestinal Vasculature Decreases after Culture in Controlled Flow Conditions. **(A)** Representative images of FITC-Dextran (green in i-iii) following perfusion through the intestinal vasculature after 1 h (i), 24 h (ii), and 48 h (iii) of tissue culture with controlled flow. Regions are labeled as to their location relative to the occlusion site (O), with more distal areas labeled as Far Left (FL) and Far Right (FR) and more proximal areas as Close Left (CL) and Close Right (CR). Scale bars in each image (i-iii) are 1 cm. **(B)** Relative integrated densities of FITC-Dextran quantified in tissue after 1 h (green bar), 24 h (blue bar), and 48 h (red bar) of culture with controlled flow. Error bars represent ± standard deviation. ****p ≤ 0.0001 for 1 h vs. 24 h and 48 h. **(C)** Relative integrated densities of FITC-Dextran quantified in tissue after 1 h (green bars), 24 h (blue bars), and 48 h (red bars) of culture with controlled flow, disaggregated by region. Error bars represent ± standard deviation. *p ≤ 0.05, ***p ≤ 0.001, and ****p ≤ 0.0001 for the comparisons denoted. Biological replicates for this experiment were at a minimum of *n* = 3, and data points shown are from technical replicates across all experiments.

As an orthogonal approach to flowing fluorescent microspheres to verify capillary perfusion after experimental set up, we conducted separate experiments in which we exposed tissues to flow conditions for 1 h and then introduced FITC-Dextran to the perfusate. In all regions within these tissue preparations, we found the FITC-Dextran capable of filling capillary networks (Figure 5). We even detected this dye in regions downstream of the occluded vessel, suggesting collateral channels were capable of facilitating perfusion into regions no longer supplied by direct fluid flow. Nevertheless, after 24 h of culture under flow conditions, perfusion of intestinal capillary networks was severely limited (Figure 5), even though tissue viability was largely maintained. This decrease in microvascular perfusion and capillary access to fluid flow was even more notable at the 48 h time point, despite the lack of significant tissue degradation found by the specific quantifications applied to these tissues.

**FIGURE 6:**
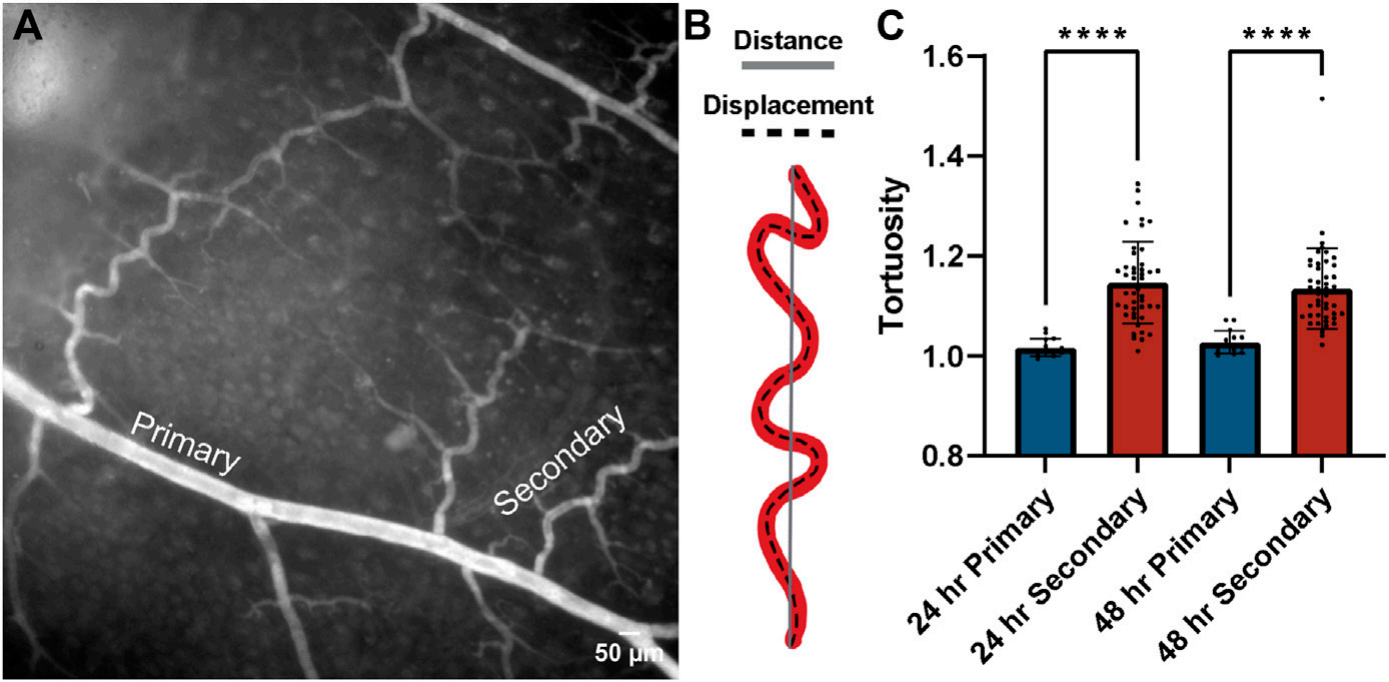
Collateral tortuosity remained increased during intestinal tissue culture in controlled flow conditions. **(A)** Representative image of intestinal tissue cultured under controlled flow conditions with primary and secondary collateral vessels denoted. Scale bar is 50 μm. **(B)** Schematic illustrating distance-metric tortuosity measurement of vessel (red) distance from end-point to end-point (solid gray line) and displacement (dashed black line). **(C)** Distance-metric tortuosity was measured at 24 h (blue bars) and 48 h (red bars) in primary (> 45 um) and secondary (< 45 um) vessels across all regions relative to the area downstream of the occlusion. ****p ≤ 0.0001 for the comparisons denoted. Tortuosity was consistently lower in primary vessels than in secondary vessels across regions after both 24 h and 48 h of tissue culture with controlled flow. Biological replicates for this experiment were at a minimum of *n* = 3, and data points shown are from technical replicates across all experiments.

Throughout these experiments, we observed changes in the morphology of collateral vessels bridging adjacent regions across the isolated tissues. Specifically, the curvature of these collateral vessels appeared to increase, which has been described in previous studies as a change in vessel tortuosity (Han, 2012; Chong et al., 2017). Increased tortuosity of blood vessels is often associated with increased intraluminal pressures and found in the context of vascular occlusive events (Hutchins et al., 1978; Jackson et al., 2005). This feature may represent a mechanism whereby intrinsic vessel geometry compensates for intraluminal pressure changes and shields the microcirculation from these changes that might otherwise damage capillaries. We therefore measured the tortuosity of primary and secondary arteries as a proxy for intraluminal pressure, asking if this pressure might decrease over time with increasing capillary access-to-flow i.e., *via* reduced resistance to flow through capillary networks. Consistent with the lack of FITC-Dextran detected in flow cultured tissues, we found a high degree of tortuosity for secondary vessels relative to primary vessels at both the 24 h and 48 h time points (Figure 6). Secondary vessel tortuosity was also fairly consistent across regions, though vessels in closer proximity to the occlusion site were slightly more tortuous at 48 h (Figure 6). By qualitative assessment, primary vessels appeared more linear, whereas secondary collaterals displayed a higher degree of inflection as a feature of their tortuosity (Sun et al., 2022). From initial observations of tortuous vessels, the flow-rate was lowered from 1.5 ml/min to 1 ml/min, but the tortuous vessels remained. Overall, these data support the idea that tissue viability was maintained in flow cultured conditions such that mechanisms (i) restricting capillary perfusion and (ii) sustaining elevated intraluminal pressure in collateral vessels remained intact over the experimental time course.

## Discussion

Tissue reperfusion outcomes vary widely following therapeutic intervention, largely due to an incomplete understanding of the underlying causes. Microvessel instability for example has been proposed as a contributing factor, with capillary-associated pericytes potentially fueling microvascular dysfunction during ischemia (Yemisci et al., 2009; Hall et al., 2014; O'Farrell et al., 2017). A broader range of experimental models are therefore necessary to identify specific factors that may limit reperfusion and in turn address important gaps in our knowledge of the “no-reflow” phenomenon (Eeckhout and Kern, 2001; Ito, 2006). To that end, we developed an *ex vivo* tissue culture model in which vessel perfusion was maintained *via* cannulation of an upstream feeder artery. We established appropriate culture conditions for this closed-loop system and verified that, following experimental set up, perfusate access to capillary beds was feasible. Tissue viability was sustained over the experimental time course, as was the stability of the microvasculature and associated pericytes. In spite of these relatively stable microcirculatory networks, perfusion rapidly became limited to larger collateral vessels, which exhibited hallmarks of a sustained elevation in intraluminal pressure, specifically an increase in their tortuosity. Taken together, our data demonstrate that, despite the lack of overt cell death and tissue or vessel degradation by the measurements applied, capillary access-to-flow was severely reduced regardless of vascular integrity (as assessed by lectin-labeling vascular networks immediately preceding flow-controlled culture, then imaging post-culture *via* confocal microscopy (See Supplemental Materials) or proximity to a primary occlusion. Though we fully acknowledge that we did not formally measure the full range of vascular integrity indicators (e.g., small molecular weight tracer leakage or tight/adherens junction assessment). The current study also highlights the utility of tissue-based platforms to complement *in vitro* and *in vivo* approaches to isolate and explore the crosstalk between cellular responses and perturbations to fluid flow conditions.

Changes in blood flow within a tissue can lead to a wide range of consequences. For instance, limited or absent perfusion results in ischemic conditions including hypoxia, and disrupts nutrient/waste exchange as well as the biophysical forces on vessel walls (Hademenos and Massoud, 1997). The duration of the ischemic event also dictates the nature of cellular responses. Hypoxic conditions, both acute and chronic, have been suggested to induce microvascular instability (Schoch et al., 2002) *via* cell death and disengagement and in turn limit the capacity for reperfusion (Galiuto et al., 2000). Pericytes for example have been described as contracting and dying in this constricted state, thereby causing regional blood flow deficits following ischemia (Yemisci et al., 2009; Hall et al., 2014; O'Farrell and Attwell, 2014; O'Farrell et al., 2017). This phenomenon may be tissue- and/or time-dependent, as our data suggest that restricted flow may occur (i) more upstream of the capillary beds where pericytes are most abundant, and (ii) independent of overt pericyte death or detachment. While pericytes may be indirectly involved in the mechanisms that limit reperfusion, we did not find extensive cell death at the 24 h or 48 h time points observed, though we did not use live imaging to establish the exact time course for these changes. Furthermore, we cannot rule out the possibility that pericytes may communicate paracrine or intracellular signals to upstream cells that may be more directly involved in vasoconstriction and limiting capillary access to flow. But we did not observe widespread pericyte death in flow-restricted regions that would suggest their involvement in reduced fluid transit. Nevertheless, future studies will be needed to build upon these observations to further elucidate the potential roles of microvascular pericytes in limiting capillary perfusion in the context of tissue ischemia and reperfusion.

An additional outcome of altered blood flow is often morphological adaptation of affected vessels. For example, in the current study, we observed fluid transit primarily through collateral channels over an acute time course. We found that these tortuous vessels were consistently present throughout the entire region receiving controlled flow, even after adjusting the flow-rate in an attempt to relieve excessive shear stress on the vessel walls. Though we did not quantify features such as bending length and inflection points (Sun et al., 2022), measuring vessel tortuosity revealed that secondary, smaller-order vessels, such as the collaterals bridging adjacent territories, saw the highest degree of tortuosity. Two possible interpretations seem to emerge from this observation. First, the increased vessel tortuosity may reflect a temporary “morphological response” to altered flow patterns, that is, an adaptation based on inherent vessel geometry as opposed to structural remodeling that likely occurs over a more extended period of time (Chappell et al., 2008; Song and Munn, 2011; Chong et al., 2017). Vessel curvature may in fact reflect a means by which higher intraluminal pressure is dissipated, such as in scenarios where flows must be reduced for functional reasons e.g., uterine spiral arteries (Osol and Moore, 2014). In our study, the degree of tortuosity being generally lower at the 48 h time-point compared to 24 h suggests that perhaps vessel tortuosity changes are an acute, functional adaptation to abnormal flow rather than a permanent, negative consequence of it. Second, the degree of tortuosity in secondary vessels being higher at 48 h only in the occluded area suggests that these vessels may be facilitating larger flow volumes than their distal counterparts (Han, 2012). These secondary collaterals may need to adapt *via* tortuosity changes more substantially as compared to non-occluded areas, which is consistent with the idea that neighboring vessels may be actively shunting flow to the occluded area as a means to sustain tissue health. Overall, vessel tortuosity observed experimentally, or perhaps even clinically, may be a useful indicator of vessel adaptations to altered blood flow states and the relative efficiency of perfusion within downstream tissues.

In addition to the data and interpretations discussed above, the work presented herein aimed to highlight the utility of *ex vivo* tissue explant platforms as complementary approaches to *in vitro* microfluidics models and *in vivo* experimentation. Vascular-focused microfluidics models (Song and Munn, 2011; Sobrino et al., 2016; Phan et al., 2017; Haase et al., 2019) are moving towards, but have yet to achieve, recapitulation of key microcirculation features including: (i) sustained capillary diameters of 10 microns or less, (ii) appropriate deposition of ECM components within the vascular basement membrane, (iii) inclusion of microvascular pericytes exhibiting *in vivo* morphologies, and (iv) network architecture and hierarchy reflecting those found in specific tissues (Hesh et al., 2019). While *in vivo* experimental models can more accurately capture the complex biological response to altered blood flow (Gonzalez et al., 2015), that complexity can make it difficult to isolate and understand the individual components involved. Activation of the inflammatory cascade (Nowicki, 2005), for example, can create challenges in understanding contributions of recruited immune cells relative to local cellular constituents. *In vivo* models are also limited with respect to the time-course of experimental interventions, as applying multiple, acute manipulations in an animal model is incredibly difficult without compromising the health and wellbeing of the subject. These models are further complicated by the broad effects that anesthesia (Luo et al., 2007), pain management (Peterson et al., 2017), and surgical intervention have on the cardiovascular and immune systems as well as on overall animal physiology. While the experimental model presented in the current study has certain limitations, this *ex vivo* tissue perfusion system may offer an intermediate approach to bridge the gap between *in vitro* and *in vivo* applications.

As with all studies, it is critical to acknowledge the limitations of our model and approaches as well as possible alternative interpretations. While *Ng2:DsRed* was used in conjuction with vessel and cellular morphology to identify pericytes, we fully acknowledge that *Ng2/Cspg4* is expressed by other cell types including other mural cells such as vascular smooth muscle cells. Another important limitation in the interpretation of our findings is that the intestinal tissue studied herein is relatively thin and may be able to absorb nutrients/oxygen from the superfusate, while tissues such as the brain are much thicker and likely be precluded from use in our experimental setup. Furthermore, interrogating potential molecular mechanisms involved in our observations was beyond the scope of the current study but will be a goal moving forward. This work provides a foundation for using this *ex vivo* tissue-based fluidics platform to transcriptionally profile vascular cell types in regions experiencing variable levels of fluid flow and mechanical stress (Culver and Dickinson, 2010). Regarding specifics for our model, it is possible that our observations may depend on the viscosity of the perfusate, which was selected to promote tissue viability but not necessarily to reflect specific characteristics of blood. Though we were able to achieve flow and intraluminal pressurization, the fluid itself may have lacked key mechanical and/or chemical properties to prevent persistent vaso-occlusion and restricted access to capillary networks. Another consideration for future studies might be the rate at which flow was reintroduced into our system. Major vessel occlusions are rapidly removed clinically to restore blood flow to downstream tissues, but we sought to reintroduce fluid flow into the cannulated tissues more moderately. Perhaps an even more gradual increase in reperfusion may limit or prevent any reactive myogenic vasoconstriction that might occur due to the increased intraluminal pressure. These and other alternative approaches and interpretations offer numerous opportunities for follow-on studies to further understand the molecular and mechanical cues that contribute to vascular adaptation to altered flow conditions and perhaps to persistent vasoconstriction events seen clinically.

## Data Availability

The raw data supporting the conclusion of this article will be made available by the authors, without undue reservation.
