# Supplementary material for: Evaluating cell viability, capillary perfusion, and collateral tortuosity in an ex vivo mouse intestine fluidics model
Source: Front Bioeng Biotechnol. 2022 Dec 9;10:1008481. doi: 10.3389/fbioe.2022.1008481 (PMC9780384; doi:10.3389/fbioe.2022.1008481)
Supplement: Supplementary file 1 [file DataSheet1.pdf]

Supplementary Materials and Methods

Detailed Materials

Mice
- NG2DsRedBAC (Stock name Tg(Cspg4-DsRed.T1)1Akik/J, #008241, Jackson Laboratory), ages 6-12 months
- B6 (Stock name C57BL/6J, #000664, Jackson Laboratory), ages 6-12 months

Dissection Tools
- Surgical scissors, forceps, tweezers, hemostat, micro probe (Roboz, catalog #RS-9422-06)
- 5mL syringe with 27G blunt tip needle
- Nylon monofilament suture, 7-0
- Vinyl dissection pads
- Tissue culture dish, 100mm x 20mm
- Surgical pins
- 33G Needles, Air-Tite Products Co., Inc., Catalog #TSK3313
- Magnetic Based Flexible Metal Arms
- PBS, 1X, without Ca, Mg, Phenol Red, sterile
- Isoflurane
- Isoflurane overdose chamber

Pump Setup
- Low-flow peristaltic pump (perfusate-pushing pump)
- Adjustable Peristaltic Dosing Pump (volume control pump)
- Silicone tubing, sizes 5mm ID x 7mm OD, 2mm ID x 4mm OD, and 1mm ID x 2mm OD
- 250mL beaker

Culture Materials
- Minimum Essential Medium
- Fetal Bovine Serum
- Penicillin-Streptomycin

Equipment
- Zeiss Axio Observer Fluorescence Microscope
- Lumen Dynamics X-Cite 120Q
- Hamamatsu Digital Camera C11440
- Zeiss LSM880 Confocal Microscope
- Nikon Spinning Disk Confocal Microscope

Detailed Methods

Preparation
- A stock solution of media is prepared by mixing 495mL of MEM with 5mL of penicillin-streptomycin. Prior to experimentation, a solution of 150mL of media with 10% FBS is prepared. Heat to 37 C until ready to use.

- Prime the peristaltic pump with the warmed media solution. Ensure all areas of the tubing are full of fluid, and that there are no air pockets present.
- Distribute 15mL of sterile PBS into a tube and keep at 37 C. 5mL is for the intracardiac perfusion, and the rest is available to be deposited onto the tissue as necessary to keep the tissue moist.

Tissue Harvest and Culture

1. The mouse is euthanized via isoflurane overdose (primary euthanasia method). The mouse is laid on its back, and ethanol is sprayed on its abdomen to control the transfer of fur to surgical tools. A vertical incision is made from the center of the lower abdomen to the base of the ribcage. Cuts are made perpendicular to the midline incision at the top and bottom to create flaps which are moved to either side to fully expose the abdominal cavity. A thoracotomy is performed (secondary euthanasia method) followed by vertical incisions on either side of the ribcage. Flipping the ribcage up exposes the chest cavity.

2. An intracardiac perfusion using 5mL of warmed PBS is performed to flush out blood.

3. The liver is pushed upward to fully expose the intestinal tract. This may be done by placing a small surgical tool such as a closed hemostat to better keep the liver separated from the lower tissue. The intestines are gently moved to the left with forceps. The cecum is identified, and a cut is made to separate the ileum.

4. The point at which the stomach and duodenum meet is identified. A cut is made to separate it.

5. The intestinal tissue is continually pulled to the left, and any areas that need to be trimmed to free the tissue from the body are identified. The tissue is fully excised by cutting connective tissue and vascular branches, particularly near other organs such as the kidneys and the bottom left of the liver.

6. The tissue is transferred into the culture dish with a centrally placed small portion of vinyl dissection padding. The two endpoints where the intestines were cut are sutured, and for each the portion of tissue between the suture and the cut is cleaned of any debris by gently squeezing it out with forceps. Care is taken to collect and remove debris from the open points of the intestines, and to clean the forceps with ethanol after this procedure.

7. An area of interest is identified based on two criteria:

a. The size of the artery. Selecting one of the largest arteries available will ease the process of needle insertion.

b. The structure of the visible downstream vasculature. The vasculature downstream of the feeder artery ideally has 2 or more distinct branch points, so that one may be occluded while the other is left as is, and comparisons may be made between the two as a result of the differing flow states they experience.

The tissue is arranged so that the area of interest is laid out on the dissection pad, while the "excess tissue" is sat behind it and out of the way. Dissection pins are used to keep the area of interest in place. The arrangement of the tissue in the culture dish is illustrated in Figure 1.

8. The dish is placed on the magnetic base holding two clamp arms. The clamp arms are attached on either side of the dish to secure it in place. Additionally, the tubing connected to the 33G needle is threaded through the space above the clamp jaws on the right arm. Keeping a portion of the tubing resting on the edge of the dish will hold the needle about level with the edge, preventing it from being inclined to shift upwards after catheterization.

9. A portion of the artery to be catheterized is isolated by gently tearing the surrounding connective tissue. Approximately 1cm of artery cleared of connective tissue is ideal. A dissection pin is placed to the left of this area, which will serve to hold the needle in place and prevent it from shifting sideways.

10. The downstream arterial branch to be occluded is identified, and the connective tissue around it is gently cleared. A suture is tied around the vessel, and pulled firmly to confirm full occlusion.

11. The neighboring, non-catheterized arteries within the area of interest are sutured. This is to simulate the resistance that would be present in these vessels in-vivo due to blood flow. Additionally, the intestine itself is sutured on either end of the area of interest, both to create resistance and to isolate the region receiving flow.

12. Returning to the artery to be catheterized, an incision is made in the middle of the isolated section. The incision should go halfway through the vessel to establish an entrance point, but not cut through it completely. A micro probe is gently inserted into the vessel to hold it open. Forceps are used to guide the

needle into the vessel, while gradually withdrawing the micro probe. Once the micro probe is fully removed, a second pair of forceps may be used to gently guide the needle further into the vessel, if necessary. The needle is advanced at least 5mm into the vessel. Successful catheterization is confirmed by pushing 1mL or less of warmed PBS. The vasculature should visibly dilate in response to flow being introduced, and no leaks should be present. Upon this confirmation, the catheter connection is secured using tissue adhesive. Any moisture in the area is soaked up using a Kimwipe folded to a point, and tissue adhesive is gently applied to the area. Care is taken to keep it contained to as small an area as possible. The adhesive is given 5 minutes to fully dry, and more is administered as needed.

12. The dish, still secured on the magnetic base, is moved into the 37 C incubator. The catheter tubing is connected to the peristaltic pump tubing, and flow is started. 50mL of the media solution is deposited into the dish, with more being added if necessary. The tissue should be fully immersed in the media.

13. The volume control pump inlet tubing is secured on the dish side wall by securing it to the left clamp arm with tape. waterproof tape. The outlet tubing is placed in the media reservoir, and this pump is turned on. An illustration of the system arrangement is shown in figure Figure 1.

  a. Within 10 minutes, the area of interest should visibly inflate, confirming that flow is traveling through the intestinal vasculature.

14. For an experiment running 48 hours, media is replenished at the 24 hour point. Media is removed entirely from the culture dish, and from the reservoir until about 50mL remains. Care is taken to leave a substantial amount of media in the reservoir so as to not allow air pockets to form in the pump tubing. Media stock solution with 20% FBS is used to replenish the reservoir and the dish, as this will be diluted with the amount of original media still remaining in the reservoir and the pump tubing.

NOTE: The primary occlusion refers to the occlusion made in a downstream branch of the canalized vessel (the vessel through which flow is administered). We qualified it as the “primary” occlusion to distinguish it from the other occlusive sutures made on the neighboring, non-canalized vessels for the purpose of simulating the resistance that would be present in vivo from blood flow. The flow is kept running throughout the duration of tissue culture. All sutures, both the primary occlusive suture as well as sutures on the neighboring arteries and on either end of the intestinal segment, also remain in place throughout the duration of tissue culture.

FITC Dextran Administration Details

The FITC Dextran size is 70 kDa (Catalog #46945-100MG-F from Millipore Sigma). FITC Dextran was administered once post-culture conditions. Thus, for a 48 hour experiment, the tissue was cultured with controlled flow for 48 hours, and then at the 48 hour point, pump-administered flow was stopped. The tissue was immediately moved to a stereo-microscope, and a single injection of 2 mL of FITC Dextran was administered through the same canalized vessel that was used for pump-administered flow.

AlexaFluor488-Lectin Labeling of Intestinal Microvessels

To characterize microvascular morphology, we utilized AlexaFluor488-conjugated lectin (ThermoFisher, Catalog #I21411) staining of intestinal vessels from *Ng2:DsRed/+* mice. For non-cultured samples and all other flow-exposed points, lectin was perfused through the vascular network immediately after the catheter was secured.

Supplemental Figures

Supplemental Figure 1. Representative images of AlexaFluor488-lectin-labeled vessels (i, v, ix, xiii; green in iv, viii, xii, xvi), *Ng2:DsRed+* pericytes (ii, vi, x, xiv; red in iv, viii, xii, xvi), and Hoechst-labeled cell nuclei (iii, vii, xi, xv; white in iv, viii, xii, xvi) in the select areas of non-cultured (i-iv) and 1 hour flow-exposed (v-viii), 24 hour flow-exposed (ix-xii), and 48 hour flow-exposed (xiii-xvi) intestinal tissues with controlled flow. Scale bars in (iv), (viii), (xii), (xvi) are 50 μm.

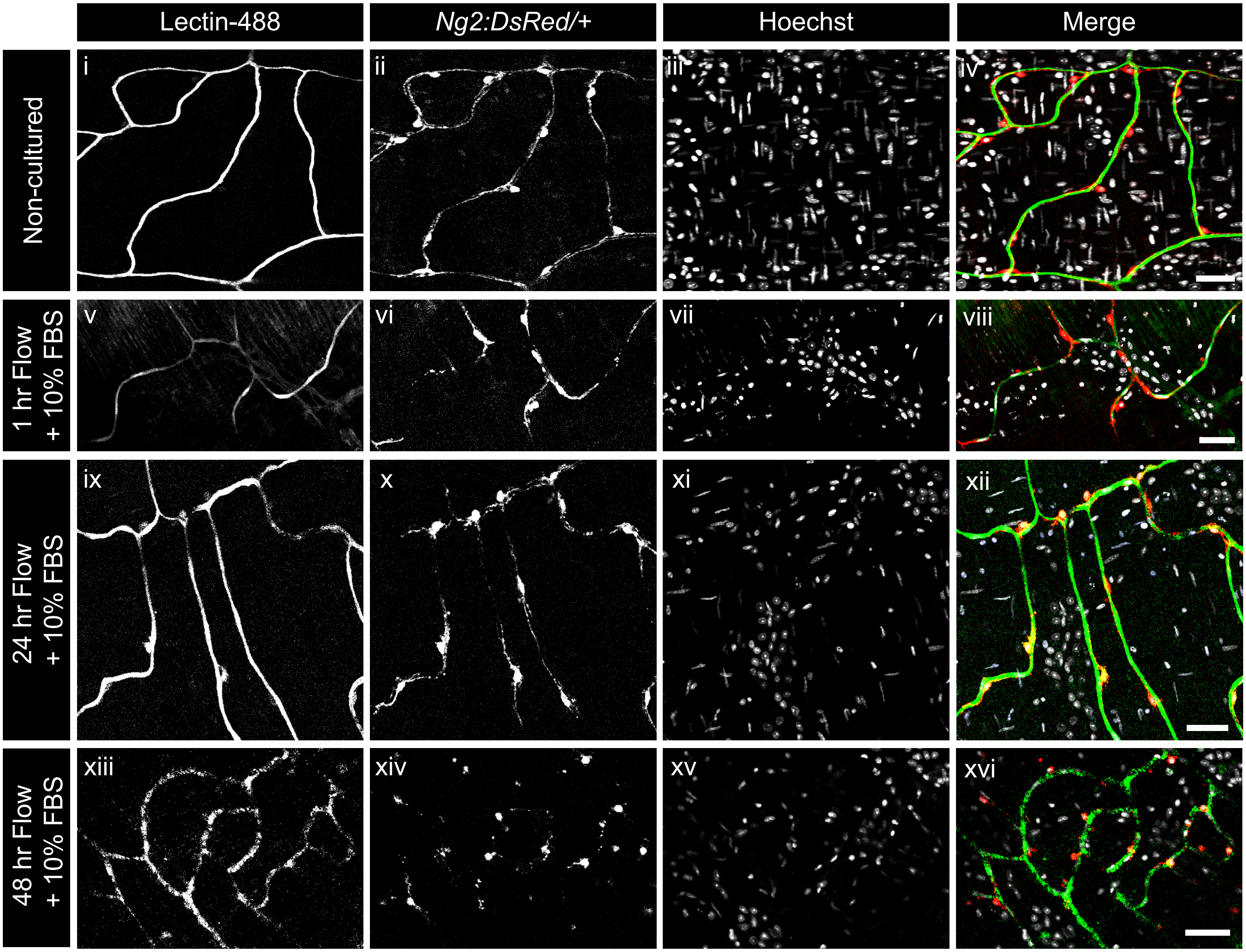

Supplemental Figure 1
